# Rethinking implementation science for health professions education: A manifesto for change

**DOI:** 10.1007/s40037-021-00688-3

**Published:** 2021-11-10

**Authors:** Aliki Thomas, Rachel H. Ellaway

**Affiliations:** 1 School of Physical and Occupational Therapy and Institute of Health Sciences Education, Faculty of Medicine and Health Sciences, McGill University, Montreal, Quebec Canada; 2 Centre for Interdisciplinary Research in Rehabilitation, Montreal, Quebec Canada; 3 Department of Community Health Sciences and Office of Health and Medical Education Scholarship, The Cumming School of Medicine, The University of Calgary, Calgary, Alberta Canada

**Keywords:** Decision-making, Implementation science, Evidence, Context

## Abstract

Implementation science approaches the challenges of translating evidence into practice as a matter of scientific inquiry. This conceptual paper uses an implementation science lens to examine the ways in which evidence from health professions education research is brought to bear on decision-making. The authors describe different decision-making contexts and the kinds of evidence they consider, and from this, they outline ways in which research findings might be better presented to support their translation into policy and practice. Reflecting on the nature of decision-making in health professions education and how decisions are made and then implemented in different health professions education contexts, the authors argue that researchers should align their work with the decision-making contexts that are most likely to make use of them. These recommendations reflect implementation science principles of packaging and disseminating evidence in ways that are meaningful for key stakeholders, that stem from co-creation of knowledge, that require or result in meaningful partnerships, and that are context specific and relevant.

**Supplementary Information:**

The online version of this article (10.1007/s40037-021-00688-3) contains supplementary material, which is available to authorized users.

## Introduction

Health professions education (HPE) research can serve many purposes including, but not limited to, influencing education practice [1]. Researchers who seek to influence education practice are frequently challenged in doing so [2]. These challenges include research evidence never making it to the right people, and research evidence being seen as lacking relevance or utility [3, 4]. In HPE there have been many calls to improve the translation of evidence into practice [5, 6], which have been linked to concepts from implementation science [7]. Implementation science employs theoretical frameworks and research methods to 1) identify the nature and magnitude of research-practice gaps; 2) identify the causes of those gaps, both individual and organizational; and 3) design and test the effectiveness of theory-driven and tailored interventions to reduce research to practice gaps [8]. However, the limited exploration of implementation science in HPE has tended to consider translation primarily as a matter of how to best expose end-users to evidence [8].

In this paper, we take a different perspective on implementation science by exploring how decisions are made in HPE. We outline how this approach can help researchers present their evidence in ways that can influence decision-making relevant to their knowledge claims (the WHAT of implementation). We end with a call to the HPE community to explore decision-informed knowledge translation [7, 8] as an implementation science approach that can better connect education scholarship to education practice (the HOW of implementation). With these objectives in mind, we present this manifesto to advance thinking on how evidence and decision-making intersect in HPE, and to call for a deeper consideration of implementation science in our field.

## Decision-making in HPE

Although research into decision-making in HPE has often focused on developing learners’ decision-making skills [9, 10], in this paper we focus instead on the decision-making that shapes educational practices within educational programs and systems. HPE is a complex and integrated undertaking that requires different levels of oversight and decision-making [11, 12], and this means that different kinds of decisions tend to be made at different levels and in different ways. Some decisions are made in, or close to educational practice (proximal), while others are made at higher levels and tend to be further from day-to-day teaching and learning (distal). While lower-level decision-making in higher education (often with a narrower scope and more proximal applications) is often relatively informal, the higher the level of decision-making in higher education (with its broader scope and increasingly distal applications), the more formal and aligned with regulation it tends to be, albeit often with less latitude to adapt and change its processes [13].

Our thesis is that research evidence in HPE that is intended to inform education practice needs to influence educators’ decisions and their decision-making processes [14]. Put another way, the form and function of HPE practices reflect the many levels and forms of decision-making. We infer from this that evidence intended to inform HPE practice should be aimed at influencing the relevant levels and forms of decision-making. We argue therefore that the utility and efficacy of evidence in HPE depends to a great extent on how well it aligns with the decision-making processes that can action its recommendations and implications. We explore this thesis by considering three aspects of decision-making in HPE: 1) levels and forms of decision-making; 2) evidence and context in decision-making; and 3) factors that compete with evidence.

### Levels and forms of decision-making in HPE

HPE programs typically have an intricate model of management and governance, both within the program and in relation to regulators and other external stakeholders [12]. For instance, curriculum committees may focus on what is taught, when it is taught, and by whom, while a visiting accreditation team may focus on gaps and discrepancies in the reporting and management of a program overall. Not only does this mean that different decisions tend to take place at different levels (i.e., individual teacher, colleague, course, program, organization, regulation, society), who is involved in decision-making, the scope of the decisions that can be made (i.e., individual, social, tactical, strategic, managerial, regulatory, sociopolitical), and how those decisions are made (i.e., committee, individual leader autonomy, workshops) can vary significantly between these levels.

To better understand this variation, we have outlined a continuum of different levels of decision-making in HPE in Tab. 1. At one end of the continuum, individual teachers typically have little say over the syllabus, curriculum, or policy as these are determined at the program level, but they typically have latitude to select or adjust their approaches to teaching and, to a lesser extent, assessment. At the other end of the continuum, regulators, professional bodies, funders, and society at large tend to have little direct influence on day-to-day teaching, but nevertheless define the broad context for educational practices and outcomes. Within this continuum there are typically several levels of decision-makers and decision-making.

**Table 1:** An outline of the continuum of decision-making levels in health professions education with examples of the scope and drivers for decision-making processes at different levels

Decision-making level	Decision-making types	Decision-making covers	Drivers of decision-making
*Individual teachers*	Individual	Primarily instruction, with limited ability to influence content, timing etc	Individual autonomy, responses to necessity and curiosity
*Teaching colleagues*	Social, discursive	Assign teaching duties, debate teaching approaches, and provide colleagues feedback	Social discussions and influences, developing shared responsibility, group norms and consensus
*Course (i.e. theme, unit)*	Tactical, limited governance	Operational details (e.g. logistical and human relations) within the parameters of the defined curriculum	Day-to-day management, responding to problems and challenges from instructors and learners, implementing policies and procedures from program and institutional
*Program*	Strategic, substantial governance	Maintaining and/or changing curriculum, syllabus, and policies and procedures; and responding to extra-program oversight	Curriculum committees, working groups, and senior managers scrutinize and set policies and procedures, and respond to program-level accountabilities (e.g. accreditation)
*Organization (school, university, hospital)*	Managerial	Setting, managing, and maintaining budgets, human resources, facilities, infrastructure, contracts, labour relations, broad policy, extramural relations	Senior leadership: education-related decisions balanced with other organizational functions and responsibilities (e.g. research, clinical, etc.)
*Regulators and funders*	Regulatory	Legitimacy and authority of programs, and broad oversight of their strategic resources and accountabilities	High-level policy (government, healthcare, professional)
*Society*	Sociopolitical	General principles, values and expectations that shape healthcare, medicine, and health professions education	Societal processes, including the media, community relations, political parties and lobby groups, donors, societal engagement, funding priorities

We can expand on this decision-making continuum by mapping out the kinds of decisions that tend to be made at different levels and the decision-making entities that are tasked with them. While specifics will likely differ between programs and systems, if researchers want to influence *what* is taught then, at least in integrated HPE programs, their evidence needs to influence course and program level decision-making. On the other hand, if they want to influence *how *the syllabus is taught, then their evidence might be better aimed at influencing individual teachers. Researchers seeking other kinds of impacts should be thinking about the decision-making processes relevant to their interests. In arguing that researchers should link their evidence to the kinds of decision-making processes in HPE that allow for its translation to practice, we have six recommendations for researchers seeking to effect or influence educational change. Our first two recommendations are that researchers:

1. Make explicit the kinds of changes they seek to bring about through the evidence they present (e.g., change instructional or assessment strategies, change policy).
2. Seek to understand how their desired changes relate to decision-making, and from this, to present their evidence in ways that can influence decision-makers at these critical levels.

We are not just proposing a linear mapping of evidence to the contexts in which it is hoped it will be influential. HPE systems tend to be ‘panarchic’ in that they involve different levels and rates of change [15]. Larger and slower levels of change (such as meeting accreditation standards or undertaking extensive curriculum change) set the conditions for smaller and faster levels (such as day-to-day teaching practice); at the same time, smaller levels (such as changing instructional strategies) can impact larger levels (such as the quality of the health professional workforce). These panarchic interactions and dependencies between decision-making levels also need to be considered. For example, higher-level HPE decisions (i.e., those from or informed by regulators, funders, professions, and society as a whole) might be expected to translate down through various levels of decision-making. However, it is more than likely that decision-making at these different levels may well reinterpret, rewrite, disregard, or dilute the original intent of these top-down directions [16].

Thus, while evidence may influence higher levels of decision-making, its translation to the day-to-day operations of HPE may be lossy. Implementation is not simply an issue of moving from ‘bench to bedside’, it is also a matter of translation between different levels of decision-making in HPE. We therefore recommend that researchers:

3. Understand the decision-making pathway(s) between the level(s) at which the decisions the evidence is intended to influence are made, and the level(s) at which these decisions will be enacted.
4. Consider how their evidence can be presented in ways that can relate to both ends of this decision-making spectrum: to decision-makers and to those implementing the decisions.

### Evidence and context in decision-making in HPE

Decision-making is inescapably context-bound, in that context shapes both the decision-making processes and the decisions that are made [17]. To that end, it is important to consider the influence of context (e.g., available resources, organizational culture, values of various stakeholders) on decision-making and on how evidence might be best (or at least better) articulated to effect change in different contexts. This might involve providing a clear and nuanced description of the context(s) in which the evidence was generated, or it might mean researchers provide vignettes of how their recommendations might be implemented in different contexts. Although all contexts are to some extent unique, we can model recurring contextual patterns to inform how evidence might be better aligned with them [18].

As an example of this, we have outlined three broad contextual patterns that can shape decision-making contexts in HPE. The first pattern focuses on the individuals responsible for the kind of change a particular piece of evidence is seeking to influence, and the kinds of decision-making processes and structures they use. For example, as we described earlier, does the evidence seek to influence policy (in which case decision-making might take place in committees) or classroom practices (in which case decision-making is more likely to lie with individual teachers)? The second pattern focuses on the cultural contexts within which change is to be affected, and whether those the evidence is seeking to influence see it as legitimate, important, and more reflective of their values and expectations. For example, one program may have a cultural disposition to resist any change (e.g., because of a will to preserve institutional values, or lack of trust between stakeholders) while another may seek to pursue change wherever possible (e.g., to demonstrate their relevance to the zeitgeist, or to improve their reputation or competitiveness). The third pattern focuses on the resources that can influence decision-making, such as the presence or lack of money, time, skills, experience, or opportunity. Evidence might be presented in ways that consider whether a proposed change or innovation is feasible in settings where money or expertise is relatively limited. Based on this, our fifth recommendation is that researchers:

5. Reflect on the different contexts in which their work seeks to have influence, and be mindful of how the structures, cultures, and resources in those contexts might create barriers or opportunities to their engagement with the evidence and what it has to offer.

### Factors that compete with research evidence in HPE

It is unfortunate that scientific evidence seldom drives decision-making in HPE [19, 20]. The reasons for this are many, but include: a perception that available evidence is of a poor quality or irrelevant; the time and effort required to access and read academic papers and extract key points; a general resistance to change; and competing priorities [19, 20]. Indeed, evidence in the form of academic publications and presentations often vies for attention with other sources of evidence. For instance, research evidence might conflict with locally generated evidence (e.g., student evaluations or accreditation results), or the opinions of different decision-makers. This is not to say that formal evidence is undervalued, rather that scientific evidence is often seen as “would be nice to consider” rather than “it must be prioritized”. In cases of competing views on the nature and the relevance of evidence, researchers must present their evidence in ways that engage with the contested and deliberative nature of HPE decision making.

We can therefore understand all decision-making in HPE as being, to some extent, contested and political. This is more likely at higher decision-making levels given the higher stakes and the wider range of competing interests, perspectives, and drivers at play. Given that decision-making in HPE often involves negotiation and compromise between different drivers and interest groups [21], our sixth recommendation is that researchers:

6. Consider how their evidence might compete with or be in conflict with other decision-making drivers or priorities. This could involve a discussion of what compromises may or may not be acceptable in implementing the evidence, or it might involve providing examples of different implementation scenarios illustrating these points. Indeed, clarification over which elements are essential and which are negotiable is a critical concern in implementation of educational innovations in general [22].

## Discussion

We drew on our direct experience of HPE in Canada and the UK, our many intersections with programs and schools around the world, and our knowledge of the field as a whole in preparing this paper. Given that the specifics vary, we might therefore consider much of the evidence that we generate as ‘middle-range evidence’ (with a nod to Merton’s concepts of ‘middle-range theory’) in that it is relevant to a particular set of contexts but not necessary to others.

We have argued that evidence seeking to influence educational practice should be targeted at the appropriate decision-making levels, stakeholders, and contexts. A systematic review of the medical literature for connections between evidence and decision-making in implementation science is beyond the scope of this paper; these connections have indeed been made, albeit in many different ways and at different levels. While individual clinical decision-making seems to dominate much of the literature, it has been observed in medicine that different kinds of decisions are made at different levels [23], that different stakeholders are involved in different kinds of decisions [24], and that evidence needs to be meaningful to them [23, 25]. In this regard, the clinical and HPE contexts are arguably similar. However, we see two major differences. Firstly, it has been argued that the evidence base for many HPE practices is less well developed than in healthcare [2]. Mapping new evidence to its relevant HPE contexts and decision-making levels early could help make it clearer what evidence is relevant in a given context. Secondly, given the stakes are often higher in healthcare practice contexts than in HPE, it is possible that the health professions educators see less of an imperative to change in the face of evidential claims they encounter [2].

Our recommendations focused on how researchers might present their evidence to better impact their target audiences. We acknowledge, however, that implementation is a broader concern and that other stakeholders can play an important role. For instance, HPE leaders and those involved at different levels of governance could be more critically engaged with the role that evidence plays in their decisions and be more vocal in helping researchers in their implementation efforts. Entities that shape the HPE research environment, namely graduate training programs, scholarly journals and conferences, and research funding agencies, could also play a more active role in aligning research with the appropriate levels of decision-making that can translate evidence into practice.

We should be clear there is no universal method or algorithm for doing this; the process is complex and probabilistic at best. Nevertheless, we can consider strategies congruent with an *integrated implementation approach *[26, 27], which requires 1) that the right stakeholders be engaged in the research process; and 2) that stakeholders be involved from the outset and throughout the research process. Guidelines on how such an integrated approach might work can be found in Tab. 1 of the Electronic Supplementary Material. We do not mean these guidelines to be prescriptive. Rather, they should serve as food for thought when engaging stakeholders in decision-making, not least because participatory and collaborative approaches must, by definition, be grounded in adaptation and tailoring. These suggestions can and should be the subject of empirical examination to test their effectiveness in enhancing decision-making. More specifically, we have proposed that researchers should seek to identify which stakeholders are involved, at which levels and for what types of decisions that are relevant to the evidence they are generating. These will be different for, say, the implementation of a new teaching strategy compared with a new admissions procedure or a curriculum overhaul. While there are different ways in which this might be approached, techniques from activity theory [28], cognitive task analysis [29], and logic modeling [30] could help in this regard. Realist inquiry with its focus on ‘what works for whom in what contexts’ could also be useful [31].

Connecting evidence to decision-making should allow for better translation and replication, as well as for understanding how the alignment between evidence and decision-making may differ across levels and contexts of decision-making. Outcome evaluation is undoubtedly the most defensible way to justify the usefulness of the approach and the resources used to affect change. This requires that key level-specific outcomes be identified early and that methods be selected that can evaluate these outcomes. Implementation science researchers have developed numerous evaluation models and frameworks [32, 33]. Tab. 2 provides recommendations for practice.

**Table 2:** Application of integrated implementation approaches to three aspects of decision-making (*DM*) in HPE

		INTEGRATED IMPLEMENTATION PRINCIPLES:	
		The right stakeholders	Authentic engagement of stakeholders in research process
		Researchers in collaboration with a local stakeholder/champion should:	
*General principles*		Identify and engage the right stakeholders for the evidence that is being implemented and its optimal point(s) of influence Make sure stakeholder engagement is meaningful, not tokenistic and/or only meeting researcher needs Ensure transparency and accountability in stakeholder selection	Engage stakeholders as early in the research process as possible Ensure that iterative and bidirectional feedback between stakeholders and researchers is encouraged Ensure transparency and accountability in how stakeholders are engaged Engage stakeholders in identifying target implementation audiences, what messages should be transferred, in what ways, by whom, and with what intended impacts
*Aspects of decision-making in HPE*	*Levels of decision-making*	Identify stakeholders based on the level of DM and the kinds of evidence they use in their DM Decide who else should be involved and in what ways Ensure stakeholder engagement is meaningful and valuable	Invite stakeholders to decide which stages of the research process they will participate in and how their participation will help them and the research Seek stakeholder feedback at every stage on how the research relates to DM and how it might be adjusted to be more relevant to decision-makers Enable stakeholder participation through supports, incentives, and/or recognition meaningful to them Collaborate in designing and executing a knowledge translation strategy that align with their DM processes
	*Context of decision-making*	Engage stakeholders from the contexts from which the evidence was generated and where the evidence will be implemented Explore how contextual variation is (or might be) seen by stakeholders as a factor in who is involved in DM and how	Encourage stakeholder feedback from a range of similar appropriate DM contexts at each stage of the research to account for contextual variation. Explore with stakeholders how contexts can change the DM implications of the research Explore research limitations with stakeholders Design and adjust knowledge translation activities to be meaningful and accessible in different contexts and to reflect the needs and dynamics of different and evolving DM contexts
	*Factors that compete with evidence*	Select stakeholders who understand how priorities are set and conflicts are resolved in DM Engage stakeholders with varying conceptions of evidence and its legitimacy in DM processes Explore the nature of the evidence that may be contested and how competing priorities can be resolved Identify and manage conflicts of interest between researchers and stakeholders	Engage stakeholders in exploring how competing priorities might constrain knowledge translation activities and how the research design and execution might be adapted to be more useful and compelling in informing DM Engage stakeholders in ensuring that knowledge translation activities are meaningful, accessible, tractable, and practical for decision-makers when faced with competing priorities

Each stage in the research process is an opportunity for significant collaboration with stakeholders at all levels including the development or refinement of the decision that needs to be made, identification of DM processes, enactment of decision, monitoring of the process of DM and evaluation of the outcomes, crafting of the message and dissemination of the DM outcomes. This engagement is predicated upon HPE researchers’ ability to garner trust from stakeholders at different levels in the DM continuum and to demonstrate their leadership in committees, initiatives and research networks

We have outlined key aspects of decision-making in HPE and the ways in which the connections between evidence and its impact can be developed. We have also been clear that these are not causal conditions; rather, that they will help to make translation to practice and influence on decision-making from evidence more likely.

We also note that there are practical and conceptual limits to which researchers can engage stakeholders, both in variety and scale. What a proportionate level of engagement and alignment will look like will depend on the nature of the evidence generated by the research, the kinds of impacts that are being sought, the kinds of decision-making contexts involved, and the resources (e.g., time) available to all concerned. We also acknowledge that the additional effort and expertise required in mapping evidence to its decision-making contexts and dynamics suggests we need further research into this topic alongside training for our researchers in implementation science techniques.

Contributing to organizational change may be a relatively new concept to researchers but it is, arguably, what leaders in HPE must frequently do. We are therefore in part advocating for more substantial and deliberate scholar-leadership; the leadership and organizational literature may help in this regard, whether it is the work of change scholars such as John Kotter [34] or those who directly explore decision-making [35].

We also acknowledge that not all researchers want to effect change, or at least not to effect specific programmatic changes. There is, after all, a tension in our field between communications aimed at other researchers and those aimed at influencing practice—even in an applied field, scholarly communications can vary reasonably and target other researchers [36]. Either way, the argument for understanding and targeting an audience still applies, especially in regard to decision-making. While the need for researchers to influence decision-making is not new [14], we have used an implementation science lens to argue for ways in which this gap can be closed and have made explicit the differences in the levels and kinds of decisions that are made in HPE.

Finally, we have presented a thesis that, while drawn from direct and indirect experiences and knowledge of the field, has not been rigorously tested in practice. We fully acknowledge that more research is needed to explore how our manifesto itself translates to practice.

By providing evidence of how to make decisions at different levels and with different actors, and by considering the consequences of different decisions and decision-making processes, we may find ourselves faced with a whole new science, a science of HPE decision-making. Our hope then is to generate a discourse on implementation science, one that considers actors, levels, culture, and compromise. In the absence of such a discourse and well thought out research agenda, our attempts at moving the science of decision-making forward, will be fragmented at best. Scholars [5, 20] have planted the seeds for future empirical work and discussion of implementation science and evidence-informed HPE. We invite others to join us in making this manifesto a reality.

## Supplementary Information

**Table 1** Application of integrated implementation approaches to three aspects of decision-making (DM) in HPE
