# Supplementary material for: Rethinking implementation science for health professions education: A manifesto for change
Source: Perspect Med Educ. 2021 Nov 10;10(6):362–8. doi: 10.1007/s40037-021-00688-3 (PMC8633355; doi:10.1007/s40037-021-00688-3)
Supplement: Supplementary file 1 — Table 1 Application of integrated implementation approaches to three aspects of decision-making (DM) in HPE [file 40037_2021_688_MOESM1_ESM.docx]

**Electronic Supplementary Material**

**Table 1** Application of integrated implementation approaches to three aspects of decision-making (DM) in HPE

|  | | **INTEGRATED IMPLEMENTATION PRINCIPLES** | |
| --- | --- | --- | --- |
|  |  | **The right stakeholders** | **Authentic engagement of stakeholders in research process** |
|  | | Researchers in collaboration with local stakeholder/champions should: | |
|  | **General principles** | Identify and engage the right stakeholders for the evidence that is being implemented and its optimal point(s) of influence  Make sure stakeholder engagement is meaningful, not tokenistic and/or only meeting researcher needs  Ensure transparency and accountability in stakeholder selection | Engage stakeholders as early in the research process as possible  Ensure that iterative and bidirectional feedback between stakeholders and researchers is encouraged  Ensure transparency and accountability in how stakeholders are engaged  Engage stakeholders in identifying target implementation audiences, what messages should be transferred, in what ways, by whom, and with what intended impacts |
| **ASPECTS OF DECISION-MAKING IN HPE** | **Levels of decision-making** | Identify stakeholders based on the level of DM and the kinds of evidence they use in their DM  Decide who else should be involved and in what ways  Ensure stakeholder engagement is meaningful and valuable | Invite stakeholders to decide which stages of the research process they will participate in and how their participation will help them and the research  Seek stakeholder feedback at every stage on how the research relates to DM and how it might be adjusted to be more relevant to decision-makers  Enable stakeholder participation through supports, incentives, and/or recognition meaningful to them  Collaborate in designing and executing a knowledge translation strategy that align with their DM processes |
|  | **Context of decision-making** | Engage stakeholders from the contexts from which the evidence was generated and where the evidence will be implemented  Explore how contextual variation is (or might be) seen by stakeholders as a factor in who is involved in DM and how | Encourage stakeholder feedback from a range of similar appropriate DM contexts at each stage of the research to account for contextual variation.  Explore with stakeholders how contexts can change the DM implications of the research  Explore research limitations with stakeholders  Design and adjust knowledge translation activities to be meaningful and accessible in different contexts and to reflect the needs and dynamics of different and evolving DM contexts |
|  | **Factors that compete with evidence** | Select stakeholders who understand how priorities are set and conflicts are resolved in DM  Engage stakeholders with varying conceptions of evidence and its legitimacy in DM processes  Explore the nature of the evidence that may be contested and how competing priorities can be resolved  Identify and manage conflicts of interest between researchers and stakeholders | Engage stakeholders in exploring how competing priorities might constrain knowledge translation activities and how the research design and execution might be adapted to be more useful and compelling in informing DM  Engage stakeholders in ensuring that knowledge translation activities are meaningful, accessible, tractable, and practical for decision-makers when faced with competing priorities |

Each stage in the research process is an opportunity for significant collaboration with stakeholders at all levels including the development or refinement of the decision that needs to be made, identification of DM processes, enactment of decision, monitoring of the process of DM and evaluation of the outcomes, crafting of the message and dissemination of the DM outcomes. This engagement is predicated upon HPE researchers’ ability to garner trust from stakeholders at different levels in the DM continuum and to demonstrate their leadership in committees, initiatives and research networks.
